# Vitamin D Status and Immune Health Outcomes in a Cross-Sectional Study and a Randomized Trial of Healthy Young Children

**DOI:** 10.3390/nu10060680

**Published:** 2018-05-27

**Authors:** Neil R. Brett, Paula Lavery, Sherry Agellon, Catherine A. Vanstone, Susan Goruk, Catherine J. Field, Hope A. Weiler

**Affiliations:** 1 School of Human Nutrition, McGill University, 21111 Lakeshore Rd, Ste-Anne-de-Bellevue, Montreal, QC H9X 3V9, Canada; neil.brett@ryerson.ca (N.R.B.); paula.lavery@mcgill.ca (P.L.); sherry.agellon@mcgill.ca (S.A.); catherine.vanstone@mcgill.ca (C.A.V.); 2 Department of Agricultural, Food and Nutritional Science, University of Alberta, 4-126a Li Ka Shing Center for Health Research Innovation, University of Alberta, Edmonton, AB T6G 2E1, Canada; sgoruk@ualberta.ca (S.G.); cjfield@ualberta.ca (C.J.F.)

**Keywords:** vitamin D, immune function, children

## Abstract

In young children, the relationship between vitamin D and biomarkers of immune function is not well elucidated. The objective was to investigate relationships between vitamin D and immune function in young children. Data were from a cross-sectional study (study 1) of healthy children 1.8–5.9 years (*n* = 457) and a 12 weeks trial using vitamin D fortified foods (study 2) in healthy 1.8–8.7 years old (*n* = 77) in Montreal, Canada. Vitamin D status and ex vivo immune function were assessed. In study 1 (male: *n* = 242; 53%), plasma IL-6, TNFα and CRP were significantly higher (*p* < 0.05) in children with 25-hydroxyvitamin D (25(OH)D) ≥ 75 nmol/L compared to <50 nmol/L. In study 2 (male: *n* = 40; 52%), there were no differences in illness outcomes (duration, number of reported illnesses, etc.) among groups. In a 6–8 years old sub-group, only the peripheral blood lymphocytes were higher in the 600 IU/day vitamin D group compared to control (percent of white blood cells; control: 41.6 ± 8.0%, 600 IU/d: 48.6 ± 8.5%). IL-6 production (but not other cytokines) by isolated mononuclear cells, after ex vivo mitogen stimulation, was lower in the intervention groups compared to the control group at 12 weeks. In conclusion, in healthy young children with sufficient vitamin D status, increasing vitamin D intakes does not confer additional advantage to immune function.

## 1. Introduction

The mean vitamin D intake of Canadian children 4–8 years old (244 ± 16 IU/day) [1] are much less than the recommended intakes set to support bone health of 400 IU/day (Estimated Average Requirement: EAR) to 600 IU/day (Recommended Dietary Allowance: RDA) [2]. There is considerable evidence that vitamin D is essential for immune function (reviewed by [3]). Recent national data regarding vitamin D status shows that 20% of Canadian children had vitamin D status below the sufficient target of 50 nmol/L of serum 25-hydroxyvitamin D (25(OH)D) [4]. This is a reflection of cumulative intakes of vitamin D from exogenous and endogenous sources. To further support vitamin D status and intake guidelines for children, it is important to understand how improving vitamin D status to healthy serum 25(OH)D targets might influence immune function. 

There is a growing body of literature suggesting that immune cells primarily rely on precursor pools of 25(OH)D, instead of endocrine derived 1,25-dihydroxyvitamin D (1,25(OH)_2_D) [3,5]. This may be because the vitamin D receptor and 1,25(OH)_2_D-hydroxylase are expressed in many cells of the immune system [6,7]. Vitamin D has been demonstrated in macrophages to stimulate the production of antimicrobial peptides [8]. Higher vitamin D exposure in vitro induces a more tolerogenic immune response by upregulating regulatory T cell gene expression and affecting T cell subtypes and cytokine production [9,10].

The few previous trials that have investigated if vitamin D supplementation or food fortification affect immune function focused on outcomes related to incidence or severity of infection [11,12,13]. Specifically, one trial showed reduced acute upper respiratory tract infections (URTI) [11], whereas a recent meta-analysis of supplementation trials in children under 5 years old concluded that vitamin D intervention had no effect on incidence of illness [12]. The only previous fortified food trial in children investigating the effect of vitamin D (300 IU/day, approximating the EAR [2]) on incidence of illness observed fewer URTIs (RR 0.52; 95% CI 0.31–0.89) [13]. These studies leave an important knowledge gap in children as to other potential vitamin D related immune outcomes, including inflammatory cytokines or changes in leukocyte concentrations in blood. In healthy adults, two injections of 140,000 IU of vitamin D over eight weeks [14] or UVB exposure for four weeks [15] increased mean vitamin D status above 75 nmol/L and increased the proportion of regulatory T cells [14,15]. However, other effects of treatments are unclear due to the limited biomarker measurements [14] or possible immunosuppressive effects of UVB light [15]. Further, as reported in a meta-analysis of randomized trials in adults, vitamin D supplementation significantly decreased the circulating concentration of the inflammatory cytokine C-reactive protein (CRP) [16]. As many trials did not report vitamin D status biomarker results, the relationship between vitamin D status and CRP was unclear [16]. Thus, the primary objective was to investigate the relationship between vitamin D status and inflammatory cytokines in a cross-sectional study of healthy young children from Montreal, Canada. Based on this data, a 12 weeks randomized trial in young children in Montreal was designed to gain a more comprehensive insight into the effect of vitamin D on inflammatory markers and other biomarkers of immune function. The secondary objective was to explore how vitamin D intakes effect frequency of illnesses, white blood cell concentrations; and in a subgroup, antimicrobial peptide concentration, inflammatory cytokines, and the ability of immune cells to respond to a bacterial challenge. 

## 2. Methods

### Study Population

This study used data obtained from two studies of young children in Montreal, Canada (45.5° N latitude), conducted at McGill University. For the first objective (study 1), secondary use of data was from a representative cross-sectional analysis of 2–5 years old (*n* = 457) [17]. For the secondary objective (study 2), data was collected *a priori* in an ancillary study at baseline and at 12 weeks in 2–8 years old (*n* = 77) children enrolled in a 12 weeks randomized, controlled trial (ClinicalTrials.gov: NCT02097160) [18]. A subset of buffy coat samples from 6–8 years old (*n* = 22) enrolled in this trial were used to explore the relationship between vitamin D status and biomarkers of immune function under the stimulus of an antigen. These studies and analyses, were approved by the McGill University Faculty of Medicine Research Ethics Board (Study 1 secondary data analysis IRB number: A07-M58-09B, Study 2 IRB number: A10-M111-13A) in accordance with the Tri-Council policy on ethics [19] and informed consent from all parents or legal guardians was obtained.

## 3. Study 1

This cross-sectional study occurred between June 2010 and June 2011 in a random sample of daycares (*n* = 77, which is 10% of all daycares: *n* = 733) licensed with the Ministère de la Famille et des Ainés, representing 91% of the regions in Greater Montréal. The recruitment of children (*n* = 534) was proportionally distributed across seasons. Inclusion criteria included healthy term born children 2 through 5 year of age. Exclusion criteria included diseases associated with disturbances of bone metabolism, known or suspected serious chronic illness of childhood, use of medications known to affect bone metabolism in the past three months, history of prior treatment for vitamin D deficiency and severe anemia. 

### 3.1. Assessments

#### 3.1.1. Blood Sampling, Vitamin D Status, and Immune Outcomes

Children were non-fasted and had 1 mL capillary blood samples taken via finger lance (0700 h–1200 h). Samples were collected into heparinized vacutainers and plasma stored at −80 °C for <12 months until analysis. Total 25(OH)D was measured using a chemiluminescent immunoassay on an autoanalyzer (Liaison, Diasorin). The sensitivity of the assay was 10 nmol/L for 25(OH)D. The inter- and intra-assay CVs were <7.5% using Diasorin controls and National Institute for Standards and Technology 25(OH)D standards 972a level 1 and 4. Accuracy was 96% using the midrange of the manufacturer’s specifications. The laboratory also maintains certification with the Vitamin D External Quality Assessment Scheme (DEQAS). Plasma CRP (CV 6.7%, assay range: 0.8–50 ng/mL), IL-6 (CV 10.4%, assay range: 3.1–300 pg/mL), and TNFα (CV 9.8%, assay range: 15.6–1000 pg/mL) were measured using manual ELISA kits (R&D Systems Quantikine, USA). To only include healthy participants, data from children was removed from the analysis if CRP was >10 mg/L.

#### 3.1.2. Dietary Assessment, Demographics and Anthropometry

Twenty-four hour food intake assessments were used to assess macronutrient and energy intake as previously reported [17,18]. It has been shown that more than seven days are needed to estimate micronutrient intake [20]. Therefore, a validated 13-item semi-quantitative 30-day food frequency questionnaire (FFQ) was used to estimate vitamin D and calcium intakes [17]. The FFQ and 24-h assessment were completed by the parents with the assistance of a registered dietitian. Nutrient intake was generated using Nutritionist Pro™ (Axxya Systems LLC, Stafford, TX, USA) and the Canadian Nutrient File version 2010b. 

Survey data was acquired for self-reported income, ethnicity, and education. Height was measured using a portable stadiometer (Seca 216, Seca Medical Scales and Measuring Systems, Hamburg, Germany) to the nearest 0.1 cm. With the child wearing light clothing and no shoes, body weight was measured to the nearest 0.5 kg using a balance-beam scale (Detecto, Webb City, MO, USA). BMI (kg/m^2^) was then calculated and *Z*-scores for height, weight and BMI were calculated using the WHO 2007 growth standards/references for children (WHO AnthroPlus, Geneva, Switzerland).

### 3.2. Statistical Analyses

The sample size for the cross-sectional study was estimated based on obtaining *n* > 500 proportionally by season, from a random sample of 10% (*n* = 77) of licensed daycares (*n* = 733) in Greater Montreal. All data entry was double audited and tested for normality using the Kolmogorov-Smirnov test and homogeneity of variance using the Bartlett test. Data analyses were conducted using SAS (version 9.3, SAS Inst. Cary, NC, USA). Spring was defined as 20 March–20 June, summer as 21 June–22 September, fall as 23 September–21 December, and winter as 22 December–19 March. 

For looking at differences among vitamin D status groups (<50 nmol/L, 50–74.9 nmol/L, ≥75 nmol/L), a mixed model ANOVA was used accounting for fixed effects (sex and age strata) and random effects (e.g., demographics, season etc.) with post-hoc testing where necessary (i.e., interactions) using Bonferroni correction. Non-parametric data were log transformed and parametric analysis used. Data are presented as mean (SD) or median (IQR) depending on normality for continuous data or as proportions for ordinal data (e.g., sex). A *p*-value <0.05, after adjustment for multiple comparisons where applicable, was accepted as significant.

## 4. Study 2

For the 12 weeks trial (study 2), recruitment was in January and February 2014. There is limited UVB solar radiation from October to early April above 40° N latitude [21]; the study was planned to run from January to April to avoid UVB exposure as a source of vitamin D. However, 22 participants had their 12 weeks study visit in early May [18]. For the trial, inclusion criteria were: healthy, pre-pubertal 2 through 8 year of age, regularly consuming milk and milk products, within 2 BMI *Z*-scores from zero for age and sex based on World Health Organization (WHO) growth charts [22]; and not taking any nutritional supplements. Exclusion criteria included chronic diseases or medications known to affect vitamin D, known infections of the immune system, known anemia, or small size at birth or preterm birth < 37 weeks gestation. As described previously [18], children were randomized to one of three groups of vitamin D intake (placebo-control, 400 IU/day (EAR), 600 IU/day (RDA)) using yogurt and cheese products. 

### 4.1. Assessments 

#### 4.1.1. Blood Sampling, Vitamin D Status, and Immune Outcomes

Parents were instructed that their child could not eat anything after midnight and fasting venipuncture blood samples were taken (0700 h–1200 h). Two mL of whole blood was separated to obtain 0.5 mL of serum for measurement of 25(OH)D and another 2 mL collected into EDTA vacutainers to obtain plasma. To build upon the data from study 1, total 25(OH)D was measured using an autoanalyzer (Liaison, Diasorin, Saluggia, Italy). The inter- and intra-assay CVs were <7.5% using Diasorin controls and National Institute for Standards and Technology 25(OH)D standards 972a level 1 and 4. Accuracy was 96% using the midrange of the manufacturer’s specifications.

At baseline and 12 weeks of the trial, a complete blood count (CBC) was done using 0.5 mL EDTA whole blood (Montreal Children’s Hospital, Montreal, QC, Canada) to provide for the differential white blood cell counts. In a 6–8 y old subset of children (*n* = 22), plasma cathelicidin was measured using a commercial ELISA (HK321, Hycult Biotech, Uden, The Netherlands) with a standard curve from 0.1 to 100 ng/mL and sensitivity of 0.1 ng/mL. Also in this subset of children, peripheral blood mononuclear cells (PBMCs; from 2 mL of EDTA treated whole blood) were isolated by density gradient centrifugation over Histopaque 1077 solution (Sigma Chemial Co., St. Louis, MO, USA) as described by Field et al. [23]. PBMC were cyropreserved in 10% dimethyl sulfoxide in fetal calf serum using a slow temperature-lowering method and polyethylene vial holder (Mr. Frosty, Nalgene Labware, Lima, OH, USA) and stored at −80 °C for <12 months [24]. Samples were shipped on dry ice to the University of Alberta and methods used were similar to those described previously [25]. Lymphocytes were rapidly thawed at 37 °C and re-suspended in RPMI 1640 supplemented with 50 g/L (5 %, *v*/*v*) heat-inactivated human serum (Gibco Life Technologies, Burlington, ON, Canada). Cells were counted using a haemocytometer. Isolated lymphocytes (1 × 10^6^ cells/mL) were cultured in 1 mL complete culture medium and incubated for 72 h with and without Concavalin A (Con A; 5 ug/mL). Supernatant was collected and stored at –80 °C for later quantification of cytokines. Cytokines were measured using a multiplex electrochemiluminescence kit for 10 analytes (25 µL; Meso Scale Diagnostics, Rockville, MD, USA) that included IL-1β, IL-2, IL-4, IL-6, IL-8, IL-10, IL-12p70, IL-13, IFN-γ, and TNF-α. Immune cell subsets were identified by flow cytometry with labelled monoclonal antibodies (mAb) [26], once labelled, cells were washed and fixed in paraformaldehyde (10 g/L in PBS) and all samples analyzed (within 3 days) on the same flow cytometer (FACScan; Becton Dickinson, Sunnyvale, CA, USA). Flow cytometry analysis (30,000 cells per mAb combination) was performed on the gated mononuclear cell population.

#### 4.1.2 Dietary Assessment.

At baseline, six and 12 weeks, 24 h food intake assessments were used to assess macronutrient and energy intake as previously reported [17,18]. As described for study 1, a validated 13-item semi-quantitative 30-day FFQ was used to estimate vitamin D and calcium intakes [17]. The FFQ and 24 h assessment were completed by the parents with the assistance of a registered dietitian. Nutrient intake was generated using Nutritionist Pro™ (Axxya Systems LLC, Stafford, TX, US) and the Canadian Nutrient File version 2010b. 

#### 4.1.3. Demographics, Illnesses, UVB, and Anthropometry

Similar to study 1, self-reported income, ethnicity, and education data from surveys was collected. Parents were supplied with a validated illness questionnaire [27] containing 18 questions with four-point ordinal scales to capture information about symptoms and severity and to calculate the Canadian Acute Respiratory Infection and Flu Scale (CARIFS) score. Scores from all questions were summed, resulting in a total score between 0 (best possible health) and 54 (worst possible health) [27]. Five further questions about illness were asked to the parents to capture medications used, days missed of school, and days missed of work for parents. As previously described and reported [18], ultra-violet beta radiation exposure was ascertained qualitatively by measuring constitutive and facultative skin pigmentation at baseline and 12 weeks using a spectrophotometer (CM-700d/600d; Konica Minolta, Tokyo, Japan). Height, weight, and BMI were determined using the same methodology as described above for study 1. 

### 4.2. Statistical Analyses

The sample size for the 12 weeks trial was determined based on vitamin D status as the primary outcome and the 6–8 years old subgroup was an exploratory analysis of results in a small sample size. Similarly to study 1, data entry was double audited and tested for normality and homogeneity and data analyses were conducted using SAS (version 9.3, SAS Inst. Cary, North Carolina). For looking at differences among the three treatment groups in study 2 (control, EAR, RDA groups), a mixed model ANOVA was used accounting for fixed effects (sex and age strata, dietary group) and random effects (e.g., demographics, etc.) with post-hoc testing where necessary using Bonferroni correction. Non-parametric data were log transformed and parametric analysis used. Data are presented similarly to study 1 and a *p*-value < 0.05, after adjustment for multiple comparisons where applicable, was accepted as significant.

## 5. Results

Demographic information from studies 1 and 2 are presented in Table 1. The age range of the children from the cross-sectional study was 1.8–6.0 years with 53% male subjects, and from the 12 weeks trial the age range was 1.9–8.7 years with 52% males (Table 1). In the cross-sectional study (study 1), 27% of the children took vitamin D supplements with a median dose of 285 (IQR 130–400) IU. Furthermore, in study 1, median vitamin D intake from food was 236 (152–320) IU/day, 1% of children had vitamin D status < 30 nmol/L, 89% of children had 25(OH)D concentrations ≥ 50 nmol/L and 47% had concentrations ≥ 75 nmol/L. In the 12 weeks trial (study 2), vitamin D dietary intake was 202 (148–316) IU/day, 1% of children had vitamin D status < 30 nmol/L, 87% had 25(OH)D concentrations ≥ 50 nmol/L, and 12% had concentrations ≥ 75 nmol/L. Supplement intake was zero according to design.

**Figure 1:**
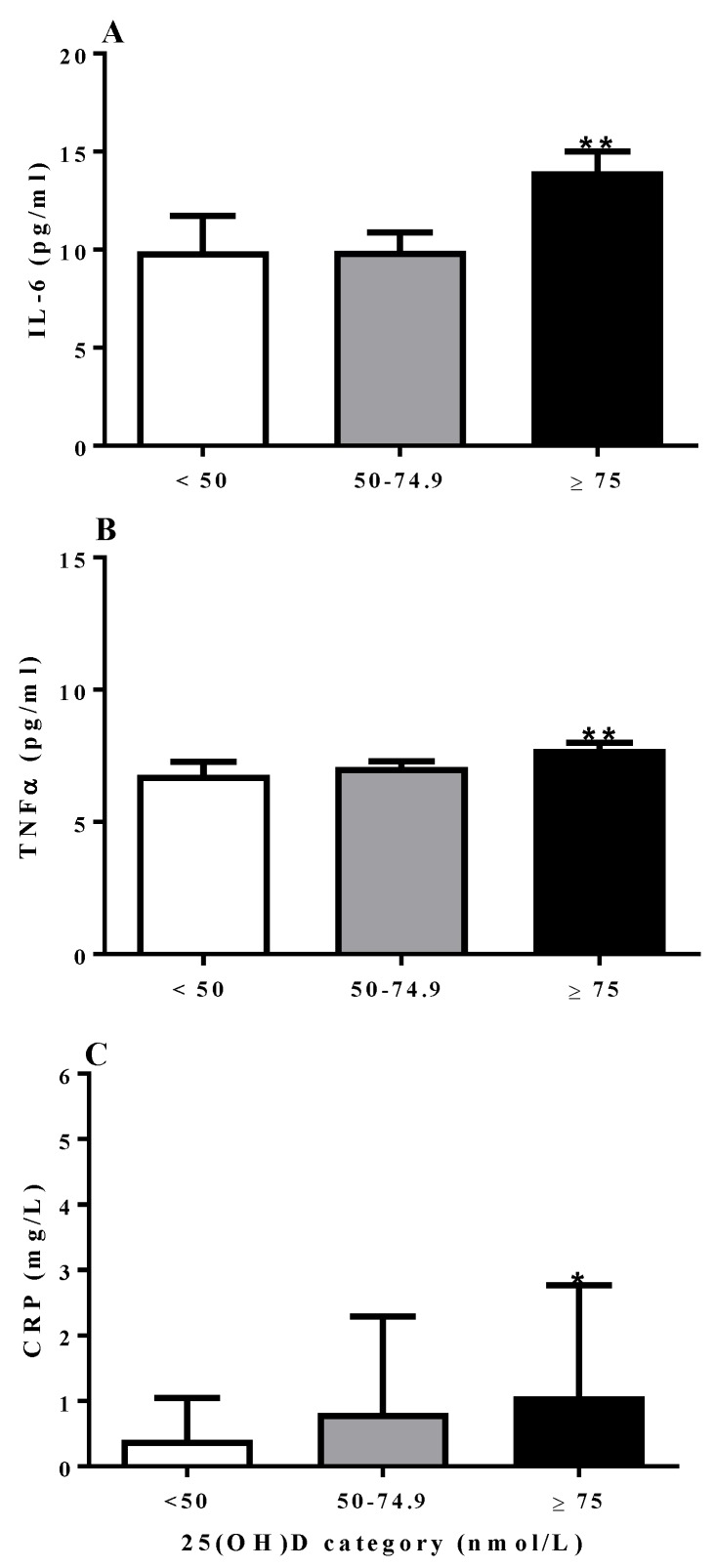
IL-6 (**A**) and TNFα (**B**) and CRP (**C**) comparison between vitamin D categories (<50 nmol/L: *n* = 52, 50–74.9 nmol/L: *n* = 189, ≥75 nmol/L: *n* = 216). Data are mean ± SD. * Significantly greater than <50 nmol/L category, ** significantly greater than <50 nmol/L category and 50–74.9 nmol/L categories (*p* < 0.05) using mixed model ANOVA, adjusted for season, age, sex, and BMI *z*-score. From a normative dataset of healthy girls 13–17 years [28], mean IL-6 and TNFα concentrations were 16.6 ± 40.8 pg/mL and 10.1 ± 21.8 pg/mL respectively. Mean CRP from a normative dataset of preschool age children was 1.9 ± 4.0 mg/L [29].

In the cross-sectional study, children measured during the summer months had the highest average vitamin D status (88.6 ± 30.0 nmol/L 25(OH)D), which was significantly higher than those measured in fall (77.4 ± 24.7 nmol/L, *p* < 0.01) and winter (70.8 ± 24.0 nmol/L *p* < 0.001), but not spring (79.7 ± 29.1 nmol/L). Children in the winter who were not taking vitamin D supplements had a mean vitamin D status of 65.4 ± 23.0 nmol/L (*n* = 77). Children with BMI *z*-scores <−1 had significantly lower plasma TNFα and IL-6 than children with BMI *z*-scores >0 (*p* < 0.01), but not children with BMI *z*-scores between −1 and 0. CRP concentrations did not differ based on BMI *z*-score or age category. Plasma IL-6 and TNFα concentration (Figure 1A,B) were significantly higher when adjusted for season, age, sex and BMI *z*-score (*p* < 0.05) for children in the ≥75 nmol/L 25(OH)D category compared to the other 2 categories. The concentrations of these two cytokines for the <50 nmol/L and 50–74.9 nmol/L categories did not differ significantly. Similarly, children with status ≥75 nmol/L, had CRP concentrations significantly higher than the <50 nmol/L category (*p* < 0.01), but not the 50–74.9 nmol/L category (*p* > 0.05; Figure 1C). 

In study 2, as previously reported [18], serum concentrations of 25(OH)D did not differ among groups at baseline (control: 58.3 ± 14.5 nmol/L, EAR: 59.2 ± 13.3 nmol/L, RDA: 60.4 ± 10.5 nmol/L). The control group was significantly different from the EAR and RDA groups at 12 weeks (control: 55.8 ± 12.3 nmol/L, EAR: 64.1 ± 10.0 nmol/L, RDA: 63.7 ± 12.4 nmol/L). During the 12 weeks, there was no significant tanning of skin from UVB solar radiation (Δ individual typological angle: 1.84 ± 3.75). Though endogenous vitamin D production from UVB exposure can occur during May, the 22 children with study follow-up sessions in May did not have more tanning of skin compared to children with April follow-ups. This was based on the changes in skin color over 12 weeks (individual typological angle; ITA) not being different between children finishing in April (2.28 ± 4.06) and May (0.85 ± 2.81). There was a total of 39 children reported contracting illnesses (colds, flus and other viruses) during the 12 weeks trial (Table 2). These 39 children (20 attended school, 14 attended daycare and five stayed at home) came from 28 families and there was no difference in vitamin D status between those children who contracted illnesses (baseline: 59.5 ± 13.4 nmol/L, 12 weeks: 62.3 ± 13.7 nmol/L) and those who did not (baseline: 60.5 ± 14.9 nmol/L, 12 weeks: 60.3 ± 10.3 nmol/L). There were no differences among groups for any illness related characteristics including duration of symptoms and CARIFS score (Table 2). 

**Figure 2:**
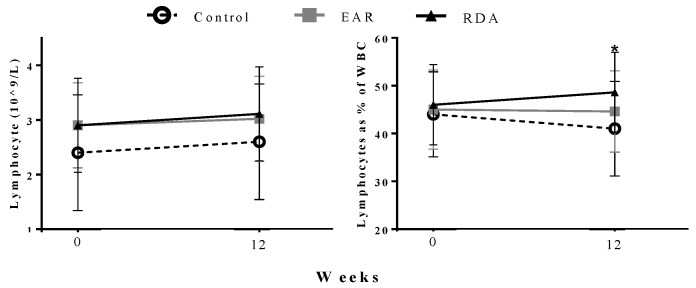
Lymphocyte concentration (reference range: 2.00–8.00 × 10^9^/L) and lymphocyte percentage of total white blood cell (WBC) concentration (reference range: 36–52%) comparison between groups from the 12 weeks trial. Data are mean ± SD using mixed model ANOVA, adjusted for age, sex, BMI *z*-score, and ethnicity. EAR: Estimated Average Requirement, RDA: Recommended Dietary Allowance. * Significantly different from control group at the same time point *p* < 0.05.

At baseline in study 2 (Figure 2) and in the 6–8 years old subset of the trial (Table 3), lymphocyte concentrations and the percentage of WBC as lymphocyte (% of WBC concentration) were not different among groups. At 12 weeks, the RDA group had a significantly higher (*p* < 0.05) percentage of lymphocytes compared to the control group when adjusted for baseline values (control: 41.6 ± 8.0 %, RDA: 48.6 ± 8.5 %) (Figure 2). In the subgroup of 6–8 years old children, this difference was more pronounced (Table 3). However, all values were within reference ranges for children. As shown in Table 3, there were no other differences in white blood cell fractions in the 6–8 years old subset of children. 

The proportion of the different mononuclear cells isolated from peripheral blood was not different among treatment groups at baseline or 12 weeks (Table 4). Production (culture concentration) of IL-2, IL-4, TNF-α by isolated peripheral mononuclear cells after stimulation ex vivo with Con A, did not differ among groups (Table 5). There was, however, a significantly lower production of IL-6 by cells from the EAR and RDA groups compared to the control group at 12 weeks (Table 5), but not at baseline (Supplemental Materials Table S1). The production of IL-6 after stimulation, did not change between 0 and 12 weeks in the control (*p* = 0.09), EAR (*p* = 0.84) or RDA (*p* = 0.23) groups. 

## 6. Discussion

These two studies address an important knowledge gap in regards to vitamin D status, inflammation and immune function in healthy young children. Providing initial exploratory data on vitamin D status and inflammation, the cross-sectional data over 1 years old suggested that vitamin D status ≥75 nmol/L is associated with higher systemic concentration of IL-6, TNFα, and CRP in children attending pre-school. A randomized trial was then designed in an effort to elucidate the effect of vitamin D on inflammatory markers, as well as many other markers of immune function including frequency of illnesses and white blood cell counts. We further explored inflammatory cytokines, the ability of immune cells to respond to a bacterial challenge and antimicrobial peptide concentration. In this winter-based trial in 2–8 years old children consuming 400 IU/day or 600 IU/day of vitamin D over 12 weeks, higher intakes of vitamin D resulted in significantly less IL-6 production by isolated mononuclear cells stimulated with Con A ex vivo, compared to the control group. However, other outcomes including, frequency of illness, antimicrobial peptide concentrations, other inflammatory cytokines and mononuclear cell phenotypes did not differ among groups. 

In the cross-sectional study (study 1), children with 25(OH)D >75 nmol/L, had higher systemic concentrations of IL-6, TNFα, and CRP. Despite being higher, plasma concentrations of these Th1 and inflammatory markers were within normative data ranges reported for healthy children (IL-6: 16.6 ± 40.8 pg/mL [30], TNFα: 10.1 ± 21.8 pg/mL [30], and CRP: 1.9 ± 4.0 mg/L [28]). CRP synthesis is stimulated by IL-6 [29], meaning elevated IL-6 may account for the observed differences in CRP. Possibly accounting for elevated IL-6 and TNFα, acute bouts of exercise (90 min) in elementary school children increased plasma IL-6 and TNFα by 125 ± 35% and 18 ± 7% respectively [31]. However, long-term increases in physical activity may relate to lower inflammatory cytokine concentrations in children [32], possibly due to lower adiposity [33]. In our cross-sectional study, though BMI *z*-score did not differ among vitamin D status categories, without measures of physical activity or adiposity, we are unable to control for these potential confounders. Though inflammatory cytokines are produced by multiple tissues, macrophages, and T lymphocytes are likely the main source of circulating concentrations [34]. More work is needed to elucidate the efficacy of 75 nmol/L as a specific vitamin D status cut-point for immune outcomes. 

To understand the significance of the effect of vitamin D on inflammatory markers, the 12 weeks trial was designed to investigate multiple other markers of immune function including illness frequency and duration. It has been shown previously that vitamin D regulates macrophage release of inflammatory cytokines and chemokines, which is thought to be a mechanism for reducing the severity of a viral illness [35]. Vitamin D status may also affect response to vaccines [36] as serum 25(OH)D was also shown to have a significant positive effect on serological response to influenza vaccination in adult cancer patients [37]. However, in the 12 weeks trial (study 2), children did not have differences in illness characteristics based on vitamin D intake or status. It is possible that this was due to the small sample size and insufficient statistical power, study length, high socioeconomic status of families, well-nourished children, or to a small number (<12%) of children with 25(OH)D ≥ 75 nmol/L [38]. In previous prospective studies of healthy children 3–15 years old, compared to those with 25(OH)D concentration ≥75 nmol/L, those with 25(OH)D <75 nmol/L reported a higher incidence of gastrointestinal and ear infections (2.05; 95% CI: 1.19-3.53) [39] and those with 25(OH)D <50 nmol/L had the highest risk of developing viral URTI (HR, 1.67; 95% CI, 1.16–2.40, *p* = 0.006) [40]. Similarly, the only previous vitamin D food fortification trial in children examining incidence of illness found that diets containing an additional 300 IU/day of vitamin D_3_, over 3 months, resulted in fewer acute URTI (RR 0.52; 95% CI 0.31–0.89) [13]. Thus, a vitamin D fortified food trial across the entire winter with a larger sample size and a wider range of vitamin D status would further clarify results of the 12 weeks trial (study 2).

As previously stated, macrophages and T lymphocytes are major contributors to circulating inflammatory cytokine concentrations [34]. Thus, white blood cell fraction concentrations and mononuclear cell phenotypes were investigated in the 12 weeks trial. Percent lymphocyte concentrations at 12 weeks in both the 12 weeks trial and the 6–8 years old subset were significantly higher in the group consuming the RDA for vitamin D. This agrees with previous work in weanling piglets, showing that vitamin D status positively associates with % total leukocytes, including monocytes and lymphocytes [41]. As % lymphocyte concentrations were within the normal range for all groups in the 12 weeks trial, and there were no among-group differences in specific mononuclear cell types in the 6–8 years old subset of children; it is unclear if a higher % lymphocyte concentration in the RDA group improved immune function. With work in healthy adults suggesting that vitamin D status >75 nmol/L promoted T cell differentiation into regulatory T cells [14,15], a larger trial in young children is needed to further elucidate the role of vitamin D in regulating immunity. 

Both studies in the present report were in healthy children. Accordingly, we were not able to study the benefits of having healthier vitamin D status during the acute phase of illness. To explore response to pathogens, ex vivo stimulation of immune cells was implemented. This is important to investigate as better local regulation of immune cell inflammatory cytokine production could result in a more effective immune response to pathogens. In the 6–8 years old subset of children from the 12 weeks trial, IL-6 production following stimulation of lymphocytes (with Con A) was significantly lower in children of the fortified groups compared to the control. With no other cytokine differences among vitamin D intake groups, it is possible that children in the control group had an increased inflammatory immune response whilst maintaining IL-2 related T cell proliferation and development [42]. Supporting this, results from in vitro studies where monocytes were similarly stimulated as those from our 6-8 years old subset, show that being pretreated with 25(OH)D (or 1,25(OH)_2_D) may inhibit an exaggerated pro-inflammatory cytokine production with the same proliferative (IL-2) response [43]. This agrees with data from men (57 years old), in a state of increased inflammation due to congestive heart failure (median TNFα concentration: 20.9 pg/mL), vitamin D_3_ supplementation decreased TNFα (median (IQR): −2.0 (−4.3, 5.5) pg/mL, *p* = 0.006) [44]. More longitudinal studies are needed to fully elucidate the physiological effects of this proinflammatory response to a T cell mitogen in children with lower vitamin D status.

Though our two studies had multiple strengths, there were several limitations. The cross-sectional design did not allow exploration of longitudinal relationships. Also, this cross-sectional study did not gather any data on previous illnesses of children, so the outcome of illness could only be investigated in the 12 weeks trial. A weakness of our 12 weeks trial was the small sample size of the 6-8 years old subset of children, meaning that the investigation was underpowered to detect small differences in infection incidence. Since we did not measure ex vivo outcomes in children 2–5 years, it is unclear if results from the 6–8 years old subset apply to 2–5 years old children in the 12 weeks trial. Also, differences in ages between the cross-sectional study and the 6-8 years old subgroup adds a limitation of combining the RCT with the cross-sectional study. The trial also had only 12% of children at baseline and less at follow up with 25(OH)D ≥75 nmol/L, meaning we could not investigate whether vitamin D status above this threshold associated with immune outcomes (like in the cross-sectional study). Since our trial was based on vitamin D fortification of foods, extrapolation to supplemental intakes is not advisable. Illness was parent-reported and not confirmed with medical records nor was the cause identified. A 12 weeks study duration may be short to detect between-group differences in severity or incidence of illness [45]. With only healthy children being included in the trial, results cannot be generalized to children born prematurely, who have obesity or other diseases that may influence vitamin D status. Further, due to inclusion criteria and the small sample size of the trial, this study is not representative of the general population. Lastly, due to the January start date and the 12 weeks duration of the trial, we were not able to measure illness or other outcomes over the entire UVB void period of the winter. 

## 7. Conclusions

In conclusion, in a cross-sectional study of young children living in an urban city at 45.5° N latitude, those with vitamin D status above 75 nmol/L appear to have significantly higher circulating concentrations of IL-6 and TNF. More trials are needed to determine whether achieving higher vitamin D status than is currently recommended (i.e., >75 nmol/L 25(OH)D) is needed for immune health. Longitudinally, vitamin D status had minimal effects on the types of immune cells in circulation. Lastly, there was an increased IL-6 production after stimulation with Con A in the control group. Future research needs to investigate cytokine and mononuclear cell outcomes related to vitamin D over the entire winter and determine the implications on the incidence and severity of infections in a larger population of young children. 

## Figures and Tables

**Table 1:** Characteristics of young children assessed for vitamin D status.

Parameter	Study 1 (Cross sectional over 1 year)	Study 2 (Baseline of 12 weeks winter trial)	
	2–5 years old	2–8 years old	6–8 years old subgroup
*n*	457	77	22
Age (years)	3.7 ± 1.0	5.1 ± 1.9	7.5 ± 0.7
	Range: 1.8–5.9	Range: 1.9–8.7	Range: 6.0–8.7
Male *n* (%)	242 (53%)	40 (52%)	12 (55%)
Ethnicity, White *n* (%)	242 (53%)	56 (72.7%)	12 (65%)
Family income: *n* (%)			
>$65,000 ^1^	264 (58%) ^2^	43 (56%)	12 (55%)
Not disclosed	37 (8%)	5 (6%)	2 (9%)
BMI *z*-score	0.50 ±1.00	0.51 ± 0.89	0.65 ± 1.09
Vitamin D intake ^3^ (IU/day):			
Total	397 (299–486)		
Without Supplements	236 (152–320)	202 (148–316)	259 (158–404)
Total 25(OH)D (nmol/L)	78.3 ± 27.3	59.3 ± 12.7	57.2 ± 11.5
Vitamin D category:			
<30 nmol/L	3 (1%)	1 (1%)	0
<50 nmol/L	51 (11%)	17 (22%)	6 (27%)
50.0–74.9 nmol/L	189 (41%)	50 (65%)	14 (64%)
75.0–124.9 nmol/L	182 (40%)	9 (12%)	2 (9%)
≥125 nmol/L	32 (7%)	0	0

Data are mean ± SD, median (IQR) or *n* (%). ^1^ Income in Canadian dollars. ^2^ Income above $60,000. ^3^ Children in the 12 weeks trial stopped taking any vitamin D supplements at least 30 days before the trial.

**Table 2:** Illness characteristics of children over 12 weeks in the winter.

	Control (*n* = 24)	EAR (*n* = 25)	RDA (*n* = 25)	Overall (*n* = 74)	*p*-Value ^4^
Number of children reported getting ill ^1^	14 (58%)	12 (48%)	13 (52%)	39 (53%)	0.68
Duration of illness (days) ^2^	4 (3–6)	5.5 (2.75–12)	3 (2.5–6)	4 (3–6)	0.17
Days of school/daycare missed ^2^	2 (1–3)	1 (1–3)	2 (1–3)	1.5 (1–3)	0.25
Days of work missed by parents ^2^	2 (1–2)	1 (1–1)	1 (1–2)	1.5 (1–2)	0.52
CARIFS score ^3^	31.1 ± 13.2	29.8 ± 12.2	39.5 ± 7.5	33.6 ± 12.1	0.07

Data are *n* (%) ^1^, median (IQR) ^2^ or mean ± SD ^3^. EAR: Estimated Average Requirement, RDA: Recommended Dietary Allowance. CARIFS: Canadian Acute Respiratory Infection and Flu Scale; 18 questions with four-point ordinal scales resulting in scores between 0 (best possible health) and 54 (worst possible health) [27]. Scores were only provided for children who were reported to have been ill. ^4^ Among-groups differences using a mixed model ANOVA accounting for age, sex, ethnicity, and BMI *z*-score.

**Table 3:** Vitamin D status and white blood cell fraction concentrations (10^9^/L) of groups from 6–8 years old subset of 12 weeks winter trial.

	0 week ^2^			12 weeks ^2^			*p*-Value	
Outcome	CTRL *n* = 7	EAR *n* = 7	RDA *n* = 9	CTRL *n* = 7	EAR *n* = 7	RDA *n* = 9	Group	Visit
25(OH)D (nmol/L)	55.0 ± 11.9	56.1 ± 9.2	59.9 ± 13.4	55.8 ± 11.9	64.1 ± 10.0*	63.7 ± 12.4 *	0.02	0.47
White blood cell fraction ^1^								
WBC (5.5–15.5 × 10^9^/L) (10^9^/L) ^3^	5.35 ± 1.07	5.42 ± 0.83	5.99 ± 1.13	5.89 ± 0.44	6.45 ± 1.20	5.45 ± 0.81	0.76	0.22
Neutrophil (1.50–8.50 × 10^9^/L) (10^9^/L) ^3^	2.45 ± 0.55	2.40 ± 0.54	2.42 ± 0.64	2.83 ± 0.70	3.16 ± 0.88	2.51 ± 0.91	0.31	0.06
Lymphocyte (2.00–8.00 × 10^9^/L) (10^9^/L) ^3^	2.30 ± 0.51	2.37 ± 0.40	2.78 ± 0.88	2.08 ± 0.36	2.55 ± 0.52	2.86 ± 0.67	0.11	0.97
Lymphocyte % of WBC (36–52%) ^3^	43.6 ± 10.2	43.9 ± 6.4	45.7 ± 8.3	37.0 ± 6.1	40.0 ± 6.3	51.7 ± 8.3 *	0.01	0.72
Monocyte (0.10–0.80 × 10^9^/L) ^3^ (10^9^/L)	0.48 ± 20.0	0.42 ± 10.1	0.43 ± 15.0	0.44 ± 13.9	0.48 ± 11.6	0.44 ± 13.0	0.85	0.75
Total CD3+ ^4^	1.6 ± 0.4	1.6 ± 0.7	1.9 ± 0.6	1.4 ± 0.4	1.7 ± 0.5	2.0 ± 0.6	0.16	0.91
Total CD4+ ^4^	0.9 ± 0.2	1.1 ± 0.3	1.1 ± 0.5	0.7 ± 0.1	1.1 ± 0.3	1.2 ± 0.5	0.13	0.84
Total CD8+ ^4^	0.6 ± 0.1	0.6 ± 0.2	0.6 ± 0.2	0.5 ± 0.2	0.6 ± 0.2	0.7 ± 0.2	0.53	0.90
Total CD25+ ^4^	0.1 ± 0.1	0.1 ± 0.1	0.1 ± 0.1	0.1 ± 0.0	0.1 ± 0.1	0.1 ± 0.1	0.97	0.35
B cells (CD19+) ^4^	0.2 ± 0.1	0.2 ± 0.1	0.2 ± 0.1	0.1 ± 0.1	0.2 ± 0.1	0.2 ± 0.1	0.31	0.71
T regulatory (CD4+25+) ^4^	0.1 ± 0.0	0.1 ± 0.0	0.1 ± 0.0	0.04 ± 0.1	0.1 ± 0.0	0.1 ± 0.1	0.67	0.83
T helper (CD3+4+) ^4^	0.8 ± 0.2	1.0 ± 0.3	1.1 ± 0.4	0.8 ± 0.1	1.0 ± 0.3	1.2 ± 0.5	0.23	0.65
T suppressor (CD3+8+) ^4^	0.5 ± 0.1	0.5 ± 0.2	0.6 ± 0.2	0.5 ± 0.1	0.6 ± 0.2	0.6 ± 0.2	0.58	0.60

^1^ Reference ranges shown in parentheses for data from complete blood cell counts. ^2^ Data are mean ± SD. ^3^ From complete blood cell counts. ^4^ Calculated using lymphocyte concentration and percentage of total gated mononuclear cells. CTRL: control, EAR: Estimated Average Requirement, RDA: Recommended Dietary Allowance, WBC: white blood cells. * Significantly different than control group (EAR: *p* = 0.01, RDA: *p* = 0.04) at 12 weeks using a mixed model ANOVA, adjusted for baseline lymphocyte % total of WBC, age, sex, and BMI *z*-score.

**Table 4:** Mononuclear cell phenotypes in 6–8 years old subset of 12 weeks trial.

	0 week			12 weeks			*p*-Value	
Cell Type	CTRL (*n* = 7)	EAR (*n* = 7)	RDA (*n* = 9)	CTRL (*n* = 7)	EAR (*n* = 7)	RDA (*n* = 9)	Group	Visit
Monocytes (CD14+)	14.4 (12.7–16.4)	13.6 (11.6–17.7)	14.8 (12.5–21.2)	14.4 (13.1–14.8)	14.9 (14.2–16.7)	16.5 (13.0–19.9)	0.10	0.84
B cells (CD19+)	9.1 (7.7–13.4)	8.0 (5.1–11.4)	6.2 (4.8–10.2)	5.4 (3.4–10.4)	6.7 5.7–9.4)	9.3 (5.7–13.5)	0.27	0.48
T Regulatory (CD4+CD25+)	2.4 (0.3–3.0)	1.9 (1.0–3.0)	2.1 (1.0–2.4)	2.5 (1.1–3.0)	2.3 (2.3–2.3)	2.5 (0.4–3.3)	0.96	0.78
T Helper (CD3+CD4+)	31.6 (26.7–34.0)	41.7 (36.4–46.4)	35.7 (29.3–37.5)	39.6 (31.9–46.7)	41.4 (38.4–42.0)	35.5 (33.3–48.2)	0.39	0.34
T Suppresser (CD3+CD8+)	18.8 (17.2–23.2)	19.8 (15.6–24.3)	21.7 (16.5–24.5)	21.6 (20.6–24.3)	21.4 (19.6–22.3)	21.0 (15.2–24.8)	0.80	0.41
CD4:CD8	1.5 (1.1–1.6)	1.7 (1.6–1.9)	1.7 (1.3–2.3)	1.7 (1.3–1.9)	1.7 (1.6–1.8)	1.7 (1.2–2.2)	0.56	0.80
Total CD3+	66.7 (58.2–71.7)	63.0 (43.8–76.1)	68.7 (59.7–74.0)	63.6 (58.8–68.7)	67.3 (63.6–71.7)	65.0 (60.3–72.8)	0.94	0.82
Total CD4+	36.2 (29.2–47.2)	42.5 (37.1–47.7)	40.1 (33.3–50.3)	34.9 (29.8–39.6)	40.1 (37.0–43.9)	37.8 (28.7–49.0)	0.31	0.54
Total CD8+	24.4 (20.7–28.6)	24.6 (22.2–28.2)	23.0 (21.0–27.1)	24.0 (20.2–27.3)	24.4 (22.2–27.6)	22.4 (20.8–25.9)	0.55	0.80
Total CD25+	4.4 (0.8–6.9)	3.4 (1.5–5.4)	3.0 (1.8–4.2)	2.9 (0.9–4.1)	2.9 (1.4–3.9)	3.2(0.5–5.3)	0.80	0.42

Data are percentage of total gated mononuclear cells and are median (IQR). No significant differences between time points or among groups using a mixed model ANOVA accounting for age, sex, ethnicity, and BMI *z*-score. CTRL: control, EAR: Estimated Average Requirement, RDA: Recommended Dietary Allowance.

**Table 5:** Cytokine production by peripheral blood mononuclear cells after stimulation with Con A and cathelicidin concentration from plasma in 6–8 years old subset at 12 weeks.

Factor	Concentration			*p*-Value	Concentration corrected for lymphocyte concentration (1 × 10^9^/L)			*p*-Value
	CTRL (*n* = 7)	EAR (*n* = 7)	RDA (*n* = 9)	Group	CTRL (*n* = 7)	EAR (*n* = 7)	RDA (*n* = 9)	Group
TNFα (pg/mL)	593 (419–959)	414 (239–455)	541 (301–1141)	0.34	345 (250–446)	126 (104–207)	190 (99–393)	0.28
IL-2 (pg/mL)	40 (39–87)	53 (30–54)	44 (12–102)	0.73	26 (17–46)	16 (13–28)	13 (4–43)	0.82
IL-4 (pg/mL)	2.8 (1.7–3.7)	2.3 (1.8–2.4)	2.0 (1.4–2.4)	0.38	1.2 (1.3–1.9)	0.8 (0.7–1.1)	0.6 (0.5–0.8)	0.19
IL-6 (pg/mL)	265 (94–422)	45* (10–56)	93* (78–176)	0.008	167 (41–249)	16 * (5–20)	30 * (25–76)	0.002
IL-10 (pg/mL)	17 (14–26)	11 (9–17)	13 (5–21)	0.80	9 (8–14)	4 (3–8)	4 (2–9)	0.35
Cathelicidin (ng/mL)	26 (17–34)	27 (25–26)	31 (25–38)	0.50	N/A	N/A	N/A	N/A

Data are median (IQR). CTRL: control, EAR: Estimated Average Requirement, RDA: Recommended Dietary Allowance, TNFα: tumor necrosis factor alpha. * Significantly different than control group (concentration: EAR: *p* = 0.006, RDA: *p* = 0.009; corrected concentration: EAR: *p* = 0.003, RDA: *p* = 0.01) using mixed model ANOVA accounting for baseline cytokine concentration, age, sex, ethnicity, and BMI *z*-score. There were no within-group changes from 0 to 12 weeks. Other cytokines not reported did not show significant differences among groups.

## Supplementary Materials

The following are available online at http://www.mdpi.com/2072-6643/10/6/680/s1, Table S1: Cytokine production by peripheral blood mononuclear cells after stimulation with Con A and cathelicidin concentration from plasma in 6-8 years old subset at baseline.
