# Supplementary material for: Vitamin D Status and Immune Health Outcomes in a Cross-Sectional Study and a Randomized Trial of Healthy Young Children
Source: Nutrients. 2018 May 27;10(6):680. doi: 10.3390/nu10060680 (PMC6024793; doi:10.3390/nu10060680)
Supplement: Supplementary file 1 [file nutrients-10-00680-s001.zip › nutrients-304243-SI.pdf]

nutrients

Supplemental Table 1. Cytokine production by peripheral blood mononuclear cells after stimulation with Con A and cathelicidin concentration from plasma in 6-8 y old subset at baseline.

	Concentration			p-Value	Concentration corrected for lymphocyte concentration ($1*10^9$/L)			p-Value
**Factor**	**CTRL (n=7)**	**EAR (n=7)**	**RDA (n=9)**	**Group**	**CTRL (n=7)**	**EAR (n=7)**	**RDA (n=9)**	**Group**
TNFα (pg/mL)	267 (171-383)	252 (17-532)	461 (172-753)	0.20	132 (78-197)	91 (8-254)	132 (67-347)	0.62
IL-2 (pg/mL)	31 (19-65)	40 (5-63)	42 (14-85)	0.70	19 (9-27)	19 (2-24)	17 (4-31)	0.84
IL-4 (pg/mL)	1.4 (1.1-2.1)	1.6 (0.2-3.1)	1.2 (1.0-2.4)	0.89	0.7 (1.4-1.2)	0.6 (0.1-1.4)	0.5 (0.3-1.1)	0.95
IL-6 (pg/mL)	44 (9-256)	12 (2-36)	49 (22-79)	0.27	22 (3-136)	5 (1-17)	14 (8-37)	0.21
IL-10 (pg/mL)	7 (5-19)	12 (1-22)	12 (8-26)	0.78	4 (2-10)	5 (0-11)	5 (2-10)	0.95
Cathelicidin (ng/mL)	25 (14-32)	27 (21-87)	43 (25-52)	0.34	N/A	N/A	N/A	N/A

Data are median (IQR). There were no differences among groups using a mixed model ANOVA accounting for age, sex, ethnicity and BMI z-score. CTRL: control, EAR: Estimated Average Requirement, RDA: Recommended Dietary Allowance, TNFα: tumor necrosis factor alpha.
